# Targeted α Therapies for the Treatment of Bone Metastases

**DOI:** 10.3390/ijms19010074

**Published:** 2017-12-28

**Authors:** Fable Zustovich, Roberto Barsanti

**Affiliations:** 1 UOC Oncologia, ULSS 1 Dolomiti, Belluno Medical Hospital “San Martino”, Viale Europa 22, 32100 Belluno, Italy; 2 Bayer Spa, Viale Certosa 210, 201156 Milan, Italy; roberto.barsanti@bayer.com

**Keywords:** radium 223, bone metastases, breast cancer, prostate cancer, tumour cell dormancy

## Abstract

The skeleton is the target tissue for many types of tumors, and, recently, the survival of patients with prostate cancer metastasis has been increased using α-emitting drugs known as targeted α therapies. The use of α-radiopharmaceuticals in medicine was hypothesized at the beginning of the nineteenth century after the observation that α-radionuclides were associated with high cell-killing energy and low tissue penetration in healthy tissues. In the prostate cancer (PC) scenario, current research suggests that this class of radiopharmaceuticals has limited toxicity, and that the mechanism of action does not overlap with pre-existing drugs, allowing us to extend therapeutic armaments and address medical oncology towards personalized and precision medicine. Ongoing studies may extend these benefits also to bone metastases deriving from other neoplasms. The aim of this review is to summarize the current research on targeted α therapies and try to identify the right patient to be treated in the right time in order to integrate in these medications in the every-day clinical practice.

## 1. Introduction

The history of α-radiation in medicine started in 1898, when Marie and Pierre Curie for the first time described the activity of Polonium and Radium, winning the Nobel Prize in physics in 1903. Later, Marie Curie received a second Nobel Prize in chemistry in 1911 for the identification and purification of radium-226 [1]. Marie Curie also evaluated the biological effects of radium-226 on human tumor cells and observed that tumor-forming cells were destroyed faster than healthy cells when exposed to radium-226 [2]. Subsequent analysis detected that emissions of radium (called α emissions) were much more lethal to tumor cells than X-rays or gamma rays, which had been previously discovered, and that the same α radiation that penetrated living tissue was less than a tenth of a millimeter in width. These seminal results were only the first step in the long journey of α radiation towards enabling valid radiopharmaceuticals to selectively destroy cancer cells in routine medical practice.

## 2. Targeted α Therapy

The evolution of modern oncology medicine during the second half of the twentieth century was most rapid in radio metabolic therapy; that is, the treatment for cancers able to selectively deliver cytotoxic radiation to cancer cells. In general, there are four types of radiation that can be used for radiometabolic therapy: β-particles, Auger electrons, conversion electrons, and α-particles; each emission is characterized by its own decay properties, linear energy transfer (LET), tissue range, half-life, and chemistry [3]. Currently, the term “Targeted Alpha Therapy” (TAT) refers to radiopharmaceutical technology that uses drugs containing radioisotopes that undergo α-particles to destroy metastatic cancer cell-diseased tissue.

**Figure 1:**
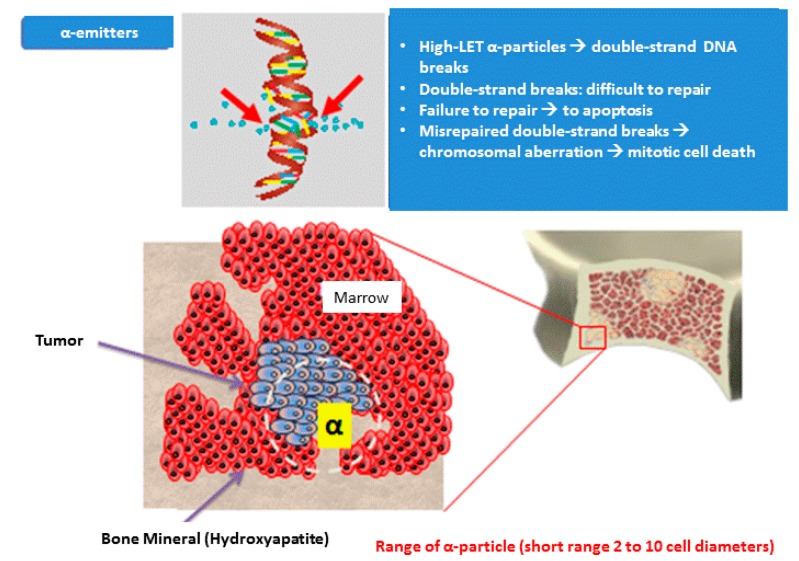
The high linear energy transfer induced by the α emitters (100 keV/µm) causes high frequencies of DNA double helix breaks in adjacent tumor cells. These are difficult to restore by cellular repair damage mechanisms, resulting in a powerful cytotoxic effect (cell death for apoptosis).

As reported in Curie’s research, α-particles have a particular advantage in targeted therapy because of their high potency and specificity. A particles are able to destroy cancer cells because their LET corresponds to 100 keV/µm [4]. This LET has a higher probability of causing DNA double strand breaks, which induce lesions in close proximity to each other that are difficult to repair without releasing cytotoxic substances into the surrounding tissues [4]. Due to this mechanism of action, α-particles have no known resistance mechanisms [4]. At the same time, α-particles limit human tissue in the very short path length below 0.1 mm, corresponding to less than 10 cell diameters (Figure 1).

According to the International Atomic Energy Agency Technical Meeting on A Emitting Radionuclides and Radiopharmaceuticals for Therapy (Vienna 2013), TATs will play an important role in the treatment of disseminated, chemo-resistant, and radio-resistant metastatic disease, against which there are no efficacious treatment options [4,5].

### 2.1. Targeted A Therapies and Skeleton

In recent times, TATs were investigated in bone metastases and the α-emitting radionuclide radium-223 is nowadays the only radiopharmaceuticals drug able to extend survival in castration-resistant prostate cancer patients affected by bone metastases [6]. The first in class radium-223 is an important opportunity in oncology: pathologic fractures and spinal cord compression are the most common consequences of bone metastases and represent significant challenges in the management of bone metastatic tumors [6].

Throughout history, the use of radiation treatment for bone metastases is correlated with external radiotherapy. This could result in pain relief in up to 70% of patients with localized pain, but the treatment is only possible for patients with multiple bone metastases and diffuse bone pain with limited extension [7]. In addition, no phase 3 study reported an increase in survival for external radiotherapy.

Radiopharmaceutical drugs containing β-emitting radionuclides (e.g., 153 samarium-ethylenediaminetetramethylenephosphonate and 89 strontium) are also used to treat pain in bone cancer patients. These drugs received recommendation from guidelines for bone-pain palliation in patients suffering from bone metastases with mainly osteoblastic lesions [8,9]. Table 1 reports the main differences between α and β-emitters used in bone metastatic tumors. A limiting problem using β-emitting drugs is the toxicity: the high radiation burden of the bone marrow may lead to a risk of significant bone marrow suppression; additionally, low LET associated with these drugs allows the survival of tumor cell without impacting on overall survival (OS) of patients [8]. Despite limitations in efficacy and tolerability, β-emitters investigations are still proceeding and new β-emitting drugs are currently being studied. For example, the experimental agent Lutetium-177-PSMA is a low-energy β-particle emitter that is chemically bonded to a monoclonal PSMA antibody. Lutetium-177 presents half-life of 6.7 days, lower killing power emissions, and longer destructive range (about 125 cells) [10]. Biophysical profile seems to indicate that this drug could induce higher levels of myelosuppression than TATs, suggesting that the use of Lutetium-177-PSMA in a clinical setting is not different to other β-emitting drugs.

### 2.2. Targeted A Therapies after Radium-226

After Curie’s discovery, researchers recognized radium-226 as the ideal treatment for cancer if this radionuclide could be transported only to the tumor and not to healthy tissues. However, an overwhelming problem was that radium-226 had a very long half-life (1601 years) and a relatively high toxicity in the body due to its decay into radioactive radon gas [11]. The consequent investigations were conducted to identify safer α-radionuclides directly acting in target site or that could be incorporated into a biological agent as radiolabeled carrier complex. For example, radium-223 presents a short half-life of 11.4 days and is able to deposit directly into bone metastases because this radionuclide has an intrinsic affinity to bone [6]. Currently, several α-particle emitters with suitable half-lives are being investigated for use in human trials. The short-lived α-emitters, astatine-211 and bismuth-213, may have potential as radio-immunotherapeutics in humans [12]. Bismuth-213 has the disadvantage of a very short (46-min) half-life, which usually requires direct injection into the tumor site. Astatine-211 is an α-emitting halogen and has an acceptable half-life for cancer therapy (7.2 h) [12]. The longer-lived α-emitters, actinium-225 (10.0 days) and thorium-227 (18.7 days), are suitable for clinical use, but differently to radium-223, the two isotopes need to be chelated and linked to monoclonal antibodies, peptides, or small molecules to achieve the biological target [13,14,15]. These radio-drugs could act differently to the common drug conjugated with antibodies, because conjugated TATs do not require cell internalization, the activity is not dependent from proliferative state of targeted cell, and the resistance has not been reported [14]. 

### 2.3. Targeted A Therapies Represent an Additional Class of Drugs in Prostate Cancer

Among the common human malignancies in developed countries, prostate cancer is the tumor with the highest incidence of bone metastases [16]; for this reason, the bone-seeking radioisotopes β-emitting and following radium-223, have been initially studied in this disease. It is worth mentioning that the use of radium-223 in this setting represents the forth therapeutic category that is able to extend survival in prostate cancer ground. Indeed, radium-223 has been demonstrated to extend survival in prostate cancer patients with bone metastases showing a non-overlapping mechanism of action and different toxicity profile than pre-existing therapies such as androgen receptor (AR) inhibitors, immunotherapy, and chemotherapy (Table 2) [17].

The next future scenario in prostate cancer will include new drugs currently in study (Table 3). With regards to TATs, the two investigational α-emitters PSMA-Thorium-227 and PSMA-Actinium-225 seem to be promising and could represent additional weapons to extend survival in the next future of prostate cancer patients.

## 3. Radium 223

Radium-223, the first α-emitter approved by the US Food and Drug Administration, is chemically similar to calcium and is incorporated into the mineral component hydroxyapatite at sites of bone turnover (e.g., bone metastases) [18]. Radium-223 primarily emits α-particles and presents a half-life of 11.4 days with decays via multiple steps before reaching its stable lead [18]. Of the total decay energy, 95.3% is emitted as α-particles, 3.6% is emitted as β-particles, and 1.1% is emitted as γ-radiation [6]. The high LET of α-emitters (80 keV/micrometer) leads to a high frequency of double-strand DNA breaks in adjacent cells, resulting in a localized antitumor effect. The α-particle range from radium-223 is less than 100 micrometers (<10 cell diameters), which minimizes damage from the surrounding normal tissue particularly to areas of marrow containing hematopoietic precursors [19]. 

### 3.1. Pivotal Studies

#### 3.1.1. ALSYMPCA (Aradin in Symptomatic Prostate Cancer)

The phase 3 trial ALSYMPCA was a randomized, double-blind, placebo-controlled study conducted in 921 patients with prostate cancer and bone metastases evaluating overall survival [6]. Secondary endpoints included time to first symptomatic skeletal event, time to total alkaline phosphatase (ALP), prostate specific antigen (PSA) progression, and total ALP response and normalization. The inclusion criteria were symptomatic CRPC, at least 2 bone metastases with no known visceral metastases (lymphoadenopathy ≤ 3 cm was allowed), and previous treatment with docetaxel or being unfit for docetaxel treatment. Patients received 50 kBq/kg of radium-223 dichloride with the best standard of care (local EBRT, corticosteroids, antiandrogens, estrogens, estramustine, or ketoconazole) or a matching placebo intravenously every 4 weeks, for a total of 6 injections. An increase in median overall survival of 3.6 months was observed in patients treated with radium-223 with the best standard of care, and this survival benefit was consistent across all subgroups, including the three-preplanned analysis (total ALP), use of bisphosphonates, and prior use of docetaxel). Significant benefits were also observed in all the secondary endpoints. The overall incidence of treatment-emergent adverse events was lower with radium-223 dichloride than with placebo (93% vs. 96%). There were no clinically meaningful differences in the rate of grade 3 or 4 hematological events between the 2 groups, and non-hematological symptoms, such as diarrhea, nausea, and vomiting, were more frequent in the radium-223 dichloride group [6]. Three-year safety analyses confirmed the lack of secondary bone tumors or hematologic malignancies in radium-223 arm [20].

#### 3.1.2. Expanded Access Program Studies

Two different Expanded Access Program studies were conducted in US (US-EAP) and in rest of the world (i-EAP). 

##### International Expanded Access Program

The international Expanded Access Program (iEAP) was a prospective, interventional, open-label, multicenter study designed to provide radium-223 to CRPC patients with symptomatic bone metastasis and to assess acute and long-term safety. The main eligibility criteria were very similar to the ALSYMPCA study; however, asymptomatic patients were also enrolled (21%) [21]. In addition, the association with denosumab and/or hormonal treatments (abiraterone and enzalutamide) was allowed. The primary outcomes of this analysis were safety and overall survival. A total of 839 patients were enrolled from 113 sites in 14 countries and 473 patients entered active follow-up. The 58% of patients in the iEAP received all 6 injections, and approximately 88% of the patients had an ECOG performance status of 0 or 1. At the time of analysis, median overall survival was 16 months, and medium time to first symptomatic skeletal events was 18 months. Grade 3/4 adverse events were reported in 38% of patients, with 21% discontinuing radium-223 due to AEs [21]. A planned post-hoc exploratory analysis showed that overall survival was, statistically speaking, significantly longer in patients with concomitant denosumab and concomitant abiraterone. Likewise, better survival levels correlated with total ALP lower than upper limit of normal, hemoglobin levels ≥10 g/dL, ECOG performance status 0, and no pain evaluated at baseline. Additionally, exploratory analyses suggested that patients with less advanced disease were more likely to receive 5–6 versus 1–4 radium-223 injections [22]. Authors concluded that use of radium-223 earlier in the treatment paradigm may allow patients to receive the full course of drug treatment [22].

##### US-Expanded Access Program

The US Expanded Access Program (US-EAP) enrolled 148 patients with baseline characteristics similar to ALSYMPCA. In this study, patients received a larger number of previous treatment than ALSYMPCA: docetaxel (60%), but also cabazitaxel (18%); abiraterone (65%); and enzalutamide (34%) [23]. In addition, greater cohort of patients in the US-EAP group had radiotherapy to prostate or prior prostatectomy. Median overall survival was 17 months, and the majority of patients had no change in ECOG PS. 

Safety profiles of patients in US-EAP were generally similar to those of ALSYMPCA, with no secondary malignancies attributable to radium-223. A majority of US-EAP patients had no change in ECOG PS. The safety profile of radium-223 was similar regardless of prior exposure to abiraterone or enzalutamide and regardless of concurrent administration of abiraterone or enzalutamide. 

Prolonged overall survival was associated with receiving 5–6 versus 1–4 radium-223 injections. Patients with less prior treatment were more likely to complete 5–6 radium-223 injections; factors predictive of receiving 1–4 versus 5–6 injections were prior abiraterone or enzalutamide, and ECOG performance status ≥ 2 and decreased hemoglobin. 

The authors concluded that radium-223 was well tolerated in the US-EAP despite these patients being heavily pretreated. In addition, using radium-223 later in the current sequencing paradigm may limit the number of patients able to receive 5 cycles of treatment [23].

### 3.2. Guidelines

Guidelines from the American Society of Oncology, European Society for Medical Oncology (ESMO), European Association of Urology, American Urological Association, and National Comprehensive Cancer Network have incorporated radium-223 as demonstrating level 1 evidence for use in CRPC patients with bone metastases without visceral metastases, who have or have not received taxane-based chemotherapy [24]. All guidelines have the highest recommendation grade and evidence level (based on ≥1 large randomized clinical trial). In 2015, ESMO developed a dynamic tool to stratify the magnitude of clinical benefits for anti-cancer therapies that assess the magnitude of drug–clinical benefits balanced against cost (ESMO-MCBS) [25]. Evaluating 80 drugs currently used in oncology, radium-223 has been identified with the highest score and currently it is the only prostate cancer drug to achieve this [25].

### 3.3. Activity on Tumor Dormancy

Although initial studies suggested the activity of radium-223 only in osteoblastic bone metastases, recent evidence indicates the activity of this radiopharmaceutical in osteolytic metastases and osteoclasts [26,27,28,29]. In fact, murine mouse models inoculated with tumor cell lines revealed that radium-223 was also able to induce double strand breaks in osteoclasts of bone metastases induced by prostate and breast cell line tumors [26,30]. Similar effects were observed in a phase 2 study done in women affected by breast cancer and bone metastases. After administration of radium-223, these patients experienced reductions in the osteoclast marker [27,28]. The activity on osteoclasts resulted in the hypothesis that radium-223 may be able to target dormant tumor cells [29]. Osteoclasts were involved in the reactivation process of tumor cells, which can assume dormancy status after the first colonization of bone tissue [31]. In fact, after initial bone colonization, prostate tumor cells can activate or remain quiescent on the basis of their interactions with the quiescent, flat-shaped osteoblasts that cover the bone surfaces (named bone lining cells). Dormancy makes these cells insensitive to the activity of drugs such as chemotherapy, so cells may remain quiescent for years without developing proliferation. The interaction with osteoclasts is one of the triggering events of the reactivation and proliferation of the dormancy tumor cell. This could display a new mechanism of action for radium-223 beyond the simple irradiation of tumor cells normally described [29].

### 3.4. Activity on Micro-Metastatic Clones of Visceral Tumor Cells

Radium-223 label suggests to exclude a priori patients with visceral metastases from radium-223 therapy because the drug does not act against soft tissue disease. A point of discussion could be whether the administration of radium-223 in patients with disease apparently limited to bones could induce a cell selection process, thus speeding up the formation of visceral metastases. Recent data reported that visceral diseases are characteristic of late stages of prostate cancer [32]. In the first phases of disease, tumors cells circulate in the blood from an initial bone metastatic site to other sites within the skeletal. Conversely, metastatic cells spreading to other tissues outside of the bone occur only in later stages; therefore, visceral spread does not necessarily originate from the primary tumors [33]. In other terms, bone triggers a dangerous cascade so that visceral metastases can originate from metastatic bone sites. According to this scenario, the administration of radium-223, especially in early stages of diseases, may induce a block in bone metastases spread and may also delay visceral dissemination. To evaluate these hypotheses, the effects of radium-223 on the development of metastases in soft tissue were studied in animal models [26]. Mice were injected with prostate cancer cell lines, were stratified accordingly to PSA levels, treated with radium-223 or control, and evaluated for the development of visceral metastases. The number of mice affected by visceral spread was generally lower in the radium-223 treatment group than the control. A greater delay in developing visceral metastases was observed, particularly in mice with lower baseline PSA levels.

### 3.5. Place in Therapy

Radium-223 is indicated for patients with castration-resistant prostate cancer, symptomatic bone metastases, and no known visceral metastatic disease. The peculiar mechanism of action of radium-223 does not seem to be tied to cross resistance mechanisms with other drugs. In fact, the mechanism of action of radium-223, which does not depend on androgen receptor pathways, is not related to the known mechanisms involved in the resistance of the other active drugs (AR-V7 splice variant) [34].

As previous reported, the current therapeutic scenario for prostate cancer includes 4 categories of drugs (chemotherapy, immunotherapy, hormone therapy, and TATs), which should be administered in sequence to ensure the best survival. The correct positioning of radium-223 in the sequence of therapeutic agents for prostate cancer is an important issue. Data achieved from ALSYMPCA and EAP studies showed that previous chemotherapy increases the risk of hematological toxicity in patients subsequently treated with Radium-223. On the contrary, no safety concerns were identified with chemotherapy following radium-223, and no detrimental effects on overall survival were observed [35]. Again, subgroup analysis of pivotal studies showed that greater survival benefits were observed in patients with less advanced characteristics observed at baseline (Hemoglobin levels, ECOG-PS, pain, and ALP). 

**Figure 2:**
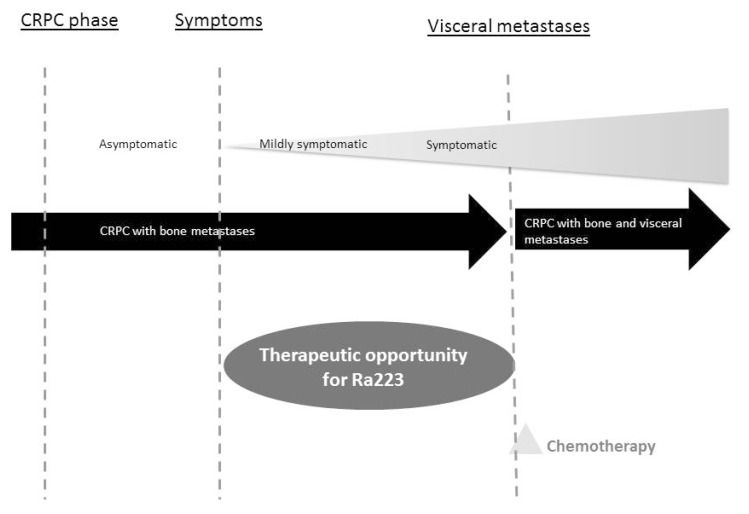
Establishing the right therapeutic collocation of available medications is crucial to prolonging the survival of patients affected by metastatic prostate cancer. Radium-223 should be administered in the early stages of the disease: pivotal studies reported that patients with a less advanced disease in terms ofECOG-*Performance Status* (ECOG-PS), hemoglobin, pain, PSA, ALP, lactate dehydrogenase (LDH), and albumin were more likely to receive 5–6 radium-223 injections. Same studies correlated the number of radium-223 injections with survival benefit. Finally, a pre-specified analysis of Aradin in SYMPtomatic Prostate CAncer (ALSYMPCA) revealed that patients who received a previous docetaxel therapy increased their risk of experiencing hematologic adverse events.

According to this evidence, the outcome of therapy can be optimized treating patients in the initial stage of disease, or in those who are minimally symptomatic and have not received prior chemotherapeutic agents (Figure 2). In contrast, delaying therapy with radium-223 to tardive lines of treatment could increase the chance of visceral metastasis, leading to a decrease in PS and increasing the risk of toxicities and the consequent risk of reducing the number of drug administrations and the survival benefit.

### 3.6. Ongoing Studies

Currently, radium-223 therapy is limited to being used as single agent in a single disease with a single scheme of administration; ongoing studies are currently evaluating new schedule and combination in prostate cancer.

A first line of studies in prostate cancer is evaluating the use of extra cycles in patients receiving a previously successful treatment with radium-223 [36]. Preliminary studies confirmed the safety of this strategy. Ongoing studies are also evaluating additional combinatorial strategies with novel anti-hormonal agents, immune-oncology agents, PARP inhibitors, and other drugs targeting ATR, ATM, and DNA-PK [37,38,39,40,41]. In addition, clinical trials are currently assessing radium-223 across a variety of osteoblastic bone-forming tumors: breast, thyroid, myeloma, and renal cell carcinoma [42,43,44,45,46]. Because preliminary studies seem to confirm the safety and efficacy of radium-223 in breast cancer patients with bone metastases [28,47,48], two separate phase II studies are ongoing. Both studies are comparing radium-223 with placebo in patients with bone predominant, HER2-negative, hormone receptor-positive metastatic breast cancer. In one of the studies, patients will receive a dose of hormonal therapy in both arms; in the other, everolimus (Afinitor) and exemestane will be administered. Assessment of symptomatic skeletal event-free survival is the primary endpoint for both studies [42]. In addition to breast cancer, a phase II study is assessing radium-223 in patients with radioactive, iodine-refractory, bone-metastatic differentiated thyroid cancer. The primary endpoint of this investigation is metabolic response, according to PERCIST criteria [43]. Additionally, a phase I study is assessing radium-223 with VEGF-targeted therapy in patients with bone-metastatic renal cell carcinoma. The primary endpoints of this study are biomarkers of osteoblast and osteoclast activity [44].

## 4. Actinium-225

Actinium-225 is an α-emitter radionuclide with a 10-day half-life [49]. TATs using actinium actinium-225 and its daughter product bismuth-213 conjugated to antibodies or carrier are promising treatments for many forms of cancer as myeloid leukemia (conjugated with anti-CD33 antibody), bladder cancer, ovarian cancer, pancreatic cancer, melanoma, and non-Hodgkin’s lymphoma. Actinium-225 conjugated to prostate-specific membrane antigen (Actinium-225-PSMA-617) is currently studied in patients with castration-resistant prostate cancer. Preclinical studies were conducted in mice and showed positive results; long-term toxicity studies indicated that late radiation nephropathy was a dose-limiting toxicity [50]. 

Actinium-225-PSMA-617 has been investigated in metastatic prostate cancer patients, having been used off-trial through compassionate access programs [51]. In early investigations, two patients achieved a complete response with regard to imaging, and no hematologic toxicity was reported, although six patients (43%) had grade 1 xerostomia and two patients (14%) had grade 2 xerostomia. 

Unpublished data announced by a recent press release described a study conducted in 80 patients of Heildelberg University [52]. The use of actinium-225-PSMA-617 involved patients who failed on multiple therapies with regard to prostate cancer and were only expected to have a median survival of 2–4 months at time of study entry. These patients received a follow-up at least of 24 weeks; in these patients, the response rate (PSA reduction and tumor shrinkage) was 75 percent and most patients were still alive 6 months after the therapy.

## 5. Thorium 227

Similarly to actinium-225, thorium-227 belongs to the actinide series of elements and results in an α-emitting radionuclide with a half-life of 18.7 days [49]. Thorium-227 decays to radium-223 (first daughter nuclide) and other short-lived radionuclides in its decay chain to stable lead-207 with α-emission of 97.4%. Dosimetry data report no significant uptake in liver or kidney [53]. Thorium-227 can be complexed by chelates conjugated to targeting moieties such as antibodies for delivery to tumor cells to create Targeted Thorium Conjugates (TTCs) [53]. 

The TTC platform is amenable to diverse targeting moieties, including antibody scaffolds or peptides. Preclinical development is underway for the treatment of breast (TTC combined with HER2 antibody), lung (TTC-EGFR), renal (TTC-CD70), ovarian, gastric, and prostate cancer [54]. A phase 1 study is ongoing and is conducted in subjects with relapsed or refractory non-Hodgkin’s lymphoma treated with thorium-227 conjugated with CD22 antibody [15]. PSMA-TTC consists of a fully human PSMA targeting IgG1 antibody covalently linked via an amide bond to a chelator moiety enabling radiolabeling with thorium-227. Unlike radium-223, which is not suitable for targeting tumors outside the bone, PSMA-TTC targeting could be extended to visceral disease [55]. In vitro cytotoxicity experiments were carried out on prostate cancer cell lines with different PSMA levels (from 3.000 to 150.000 mAbs bound/cell); in addition, in vivo biodistribution and anti-tumor efficacy were analyzed after i.v. injection of 100–500 kBq/kg to mice bearing prostate cancer xenograft models. Additionally, anti-tumor efficacy was evaluated in a PSMA expressing orthotopic bone xenograft model (LNCaP-Luc) monitored by bioluminescence imaging, micro CT, and X-ray. Statistically significant prevention of tumor growth was observed after treatment with PSMA-TTC at a dose of 100 kBq/kg in LNCaP-Luc. The promising preclinical antitumor activity of PSMA-TTC supports its development for the treatment of patients with metastatic prostate cancer [55]. 

## 6. Conclusions

One hundred years after Marie Curie received the Nobel Prize, the same radiation discovered by Curies’ was approved by international health authorities as a non-palliative drug. The daily use of radium-223 in clinical practice confirms the idea suggested in the early 1900s regarding the power and specificity of the use of α-radionuclides in oncology. Radium-223, in the current prostate cancer landscape, is not replacing chemotherapy, hormonal treatments, and other standards of care, but it is used as an additional option to prolong life and to improve quality of life. In a near-future scenario, combination therapies, including TATs with standard drugs, could allow the treatment of new patient profiles, hastening the approach to a more personalized therapy in oncology in consideration of the patient’s singular case, their stage of disease, and their preferences. Similarly, actinium-225-PSMA-617 and TTC-PSMA could fill the gap to treat very advanced patients without limiting TAT to early bone metastatic subject. Of course, the experience acquired with TATs in the prostate cancer field will be of paramount importance when assessing bone metastases in different diseases, such as breast, renal, and other tumors, when α-nuclides therapies are being studied.

## Figures and Tables

**Table 1:** Main differences between β-emitters radiopharmaceuticals and targeted α therapies.

Parametres	β-Emitters Drugs	Targeted α Therapy
Overall survival (OS) benefit	Not proven for any β-emitters in prostate cancer (PC)	European Medicines Agency (EMA), *Food and Drug Administration* (FDA) label for Radium-223
Therapy Precision	Wide Range (2–11 mm) and Low Linear Energy Transfer (LET)	Short Range (<100 μm) and high LET
Hematologic Side Effects	Dose limiting, risk of marrow ablation	limited hematological side effects
Radiation Risk Management	Often need for patient isolation due to irradiation concerns	Outpatient treatment for Radium-223

**Table 2:** Class of drugs currently available for patients with metastatic prostate cancer.

Androgen Receptor Inhibitors	Targeted α Therapy	Chemotherapy	Immuno-Therapy	Supportive Therapy
Traditional Androgen Deprivation Therapy (ADT) Abiraterone Enzalutamide	Radium-223 dichloride	Docetaxel Cabazitaxel	Sipuleucel-T	Strontium-89
				Samarium-153
				Rhenium-186
				Zoledronic acid
				Denosumab
				Steroids
Drugs with proven survival benefit				Supportive drug

**Table 3:** Main drugs with studies currently ongoing on metastatic prostate cancer.

AR Inhibitors	Targeted α Therapy	Chemotherapy	Immuno-Therapy	Supportive Therapy
ODM 201 Apalutamide	PSMA mAb Thorium-227		Pembrolizumab	Strontium-89
	Actinium-225 conjugates		Atezolizumab	
*Drugs with proven survival benefit*				*Supportive drug*
